# Successful outcome of a refractory IgA vasculitis nephritis in children treated with telitacicept

**DOI:** 10.1007/s13730-025-00983-6

**Published:** 2025-03-01

**Authors:** Yanyan Jin, Aiqin Sheng, Qian Lin, Xue He, Haidong Fu, Jianhua Mao

**Affiliations:** Department of Nephrology, National Clinical Research Center for Child Health, The Children’s Hospital, Zhejiang University School of Medicine, Hangzhou, China

**Keywords:** IgA vasculitis, Telitacicept, IgA vasculitis nephritis, Children

## Abstract

IgA vasculitis (IgAV) is the most prevalent form of vasculitis in children. While most cases of IgAV present with mild clinical symptoms and generally have a favorable prognosis, some children with IgAV nephritis may experience persistent heavy proteinuria, which is at risk of progressing to end-stage renal disease. Despite the administration of various immunosuppressive agents, treatment outcomes for these children are often suboptimal. We report the case of an 8-year-and-four-month-old girl who initially presented with rashes on both lower limbs for a duration of four days and abdominal pain persisting for two days. Renal biopsy subsequently confirmed a diagnosis of IgAV nephritis, specifically type IIIb. Despite undergoing treatment with methylprednisolone sodium succinate, cyclophosphamide, mycophenolate mofetil, leflunomide, rituximab, dapagliflozin, and other medications for over two years, her urinary protein levels remained at least 1000 mg/24 h. One month after initiating treatment with telitacicept, the patient’s urinary protein levels decreased, and two months later, they became negative. Notably, even after discontinuing immunosuppressants and glucocorticoids, the girl achieved sustained clinical remission. This case serves as a valuable clinical reference for the use of telitacicept in the treatment of refractory IgAV nephritis.

## Introduction

IgA vasculitis (IgAV) represents the most prevalent systemic vasculitis observed in childhood, with annual incidence rates ranging from 6.1 to 55.9 per 100,000 individuals across various countries [1, 2]. Renal involvement, termed IgAV nephritis (IgAVN), affects approximately 20–54% of pediatric patients with IgAV, establishing it as one of the most frequent secondary glomerular diseases in children [2]. Pediatric IgAVN is generally self-limiting, and the majority of patients exhibit favorable outcomes following symptomatic supportive treatment. Nevertheless, a minority of children may suffer from severe renal involvement, characterized by nephrotic-range proteinuria, elevated serum creatinine levels, hypertension, persistent proteinuria, and renal biopsy findings indicating more than 50% crescentic involvement [3, 4]. These children are at risk of developing long-term renal dysfunction and may progress to end-stage renal disease (ESRD), resulting in poor prognosis and necessitating aggressive therapeutic interventions [5, 6]. Prior research has indicated that among pediatric patients with moderate to severe proteinuria due to IgAVN, approximately 10–20% may advance to ESRD, with persistent proteinuria identified as an independent risk factor for unfavorable prognosis in IgAVN [5]. For those with severe IgAVN, a variety of treatment protocols are typically utilized, including intravenous pulse therapy with methylprednisolone sodium succinate (MP), mycophenolate mofetil (MMF), cyclophosphamide (CTX), azathioprine (AZA), cyclosporine A (CsA), and rituximab (RTX) [7–9]. However, there remain patients for whom multiple drug therapies are ineffective, highlighting the importance of exploring effective and safe treatment options for refractory IgAVN patients.

Telitacicept is a fully human fusion protein resulting from the combination of the TACI (Transmembrane Activator and CAML Interactor) protein with the IgG1 Fc segment. It binds to both BAFF (B cell activating factor) and APRIL (A proliferation-inducing ligand), inhibiting B cell maturation and plasma cell antibody secretion [10]. This leads to a substantial reduction in IgA1 and galactose-deficient IgA1 (Gd-IgA1) levels, as well as the formation of related autoantibodies, ultimately decreasing the deposition of glomerular mesangial immune complexes [10, 11]. In a phase II clinical trial conducted in our country involving 44 IgA nephropathy (IgAN) patients (NCT04291781), telitacicept was administered once weekly for 24 weeks. The high-dose group exhibited a 49% reduction in mean proteinuria compared to baseline, with a dose-dependent decrease in proteinuria and stable estimated glomerular filtration rate (eGFR) during follow-up. All adverse events observed during treatment were mild or moderate with no reports of serious adverse events [12]. Based on these findings, we initiated an exploration of the efficacy of telitacicept in patients with IgAVN.

## Case presentation

The patient is an eight-year-and-four-month-old female child who was admitted to the hospital on May 31, 2021, presenting with a four-day history of rash on both lower limbs and a two-day history of abdominal pain. Four days prior to admission, the patient developed a rash primarily concentrated on the calves of both lower limbs, characterized by erythematous papules elevated above the skin surface. In some areas, the rash coalesced into patches, without accompanying symptoms, such as itchiness, abdominal pain, joint swelling, or hematuria. Two days before admission, the patient experienced abdominal pain, which gradually intensified and was primarily localized around the umbilical region. There were no associated symptoms of vomiting, diarrhea, hematochezia, edema, or oliguria. The patient’s past medical history, personal history, and family history did not reveal any significant findings.

Physical examination results indicated that the heart sounds were normal, exhibiting a regular rhythm and an absence of any pathological murmurs. Breath sounds were clear in both lungs, without the presence of dry or wet rales. The abdominal region was soft, accompanied by mild tenderness in the vicinity of the umbilicus. No rebound tenderness was noted, and neither liver nor spleen was palpable. The neurological examination revealed no abnormalities. Additionally, no edema was observed throughout the body. However, dark red circular papules were visible on both lower limbs, which did not diminish upon the application of pressure.

On June 1, 2021, a routine urine test revealed microscopic hematuria accompanied by mild proteinuria, leading to the diagnosis of IgAVN. More laboratory test results are detailed in Table 1. Anti-nuclear antibody and anti-neutrophil cytoplasmic antibody were both negative. Ultrasound of the urinary system revealed no abnormalities. Gastroscopy indicated scattered ecchymoses and patchy erosions on the mucosa of the gastric antrum, duodenal bulb, and descending duodenum. During the period of hospitalization, the child exhibited significant hematuria and an elevation in the quantification of urinary protein to 2544.7 mg per 24 h. At the onset of the disease, the patient’s serum albumin level was 44.6 g/L. However, as the disease progressed, the serum albumin levels continued to decline, reaching a minimum of 26.9 g/L.

**Table 1:** Demographic and clinical baseline characteristics of patients

Characteristics	Patient	Reference range
Sex	Female	–
Age (y)	8.33	–
Height/Weight (cm/kg)	138/22.3	–
Albumin (g/L)	44.6	32.0–52.0
Serum creatinine (Enzymatic method, μmol/L)	29	21–65
WBC (× 10^9^/L)	8.93	4.0–12.0
Neutrophils (× 10^9^/L)	4.50	1.5–7.80
Lymphocytes (× 10^9^/L)	3.77	0.70–4.90
Hb(g/L)	130	110–155
PLT(/L)	290	100–400
D-dimer (mg/L)	1.37	< 0.55
IgG (g/L)	11.3	6.36–14.04
IgA (g/L)	3.6	0.63–1.79
IgM (g/L)	2.06	0.29–1.21
C3 (g/L)	1.16	0.90–1.80
C4 (g/L)	0.316	0.10–0.40
Urinary protein excretion (mg/24 h)	242.6	< 150
Urinary RBC(/HP)	12	0–5

**Fig. 1:**
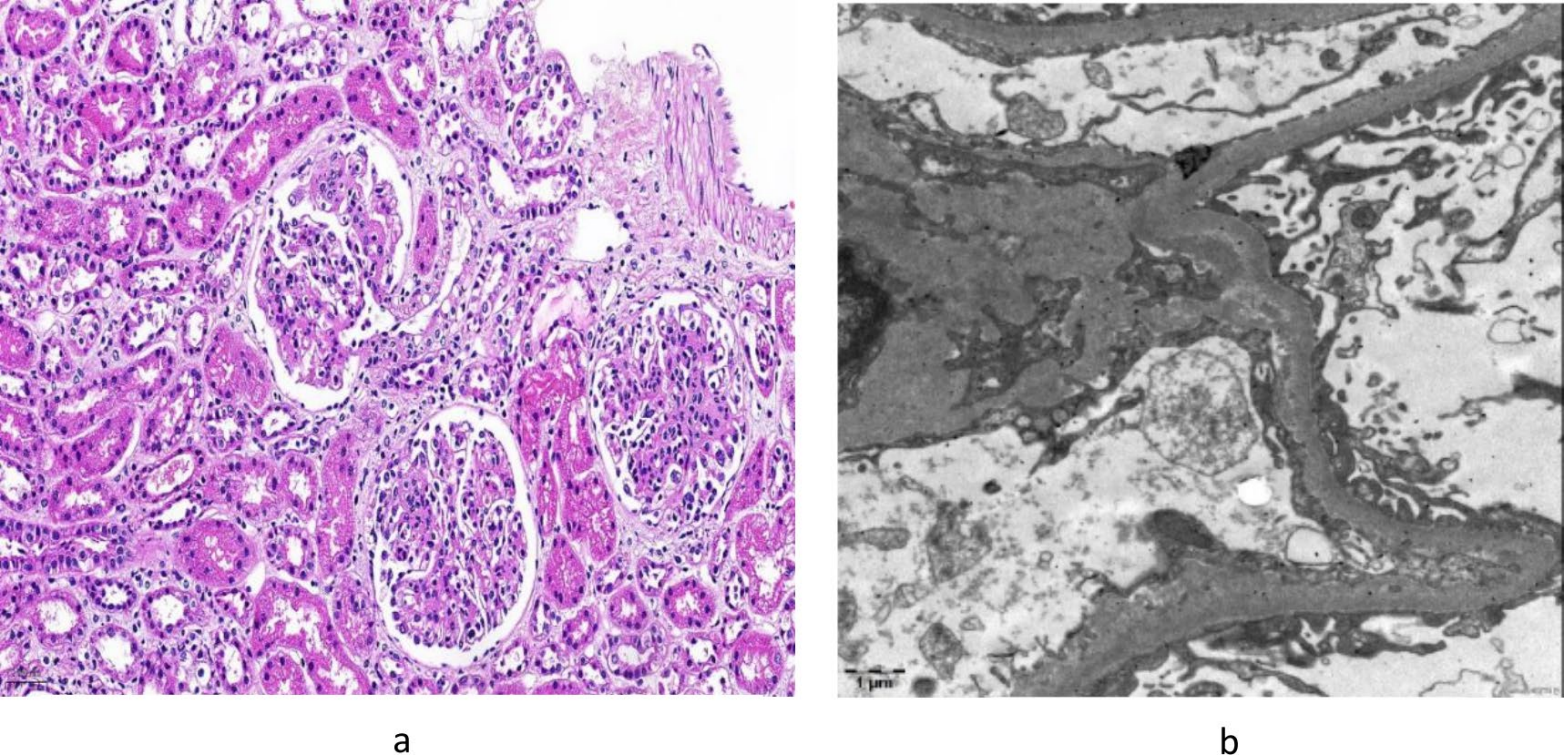
Pathological manifestations of kidney tissue under light microscopy (August 2021). **a** Mesangial cell proliferation, mild adhesion between the glomerulus and Bowman's capsule, with cellular crescents visible (HE, × 200). **b** Most foot processes fused, with electron-dense deposits seen in the mesangial area (EM)

The pathological analysis conducted following a renal biopsy on August 10, 2021 revealed mesangial cell proliferation and an increased mesangial matrix within the glomeruli with no observed thickening of the capillary basement membrane. Out of the 26 small glomeruli examined, approximately 7 exhibited mild adherence to the Bowman’s capsule, and one glomerulus examined cellular crescent bodies. There was mild granular degeneration of tubular cells, with no tubular atrophy, interstitial edema, fibrosis, scattered lymphatic mononuclear smooth muscle cells, and no interstitial blood vessels. In the frozen sections, nine small glomeruli were noted, with lgG -, lgA +  +  + , lgM + , C3 + , C4-, C1q -, and positive branching deposits of Fib + in the mesangial area. Type IV collagen detection revealed no abnormalities in the distribution of a2 and a5 chains. Electron microscopy indicated no significant thickening of the glomerular basement membrane, with most foot processes being fused, and electron-dense deposits were observed in the mesangial area, findings consistent with IgAVN type IIIb, as depicted in Fig. 1.

**Fig. 2:**
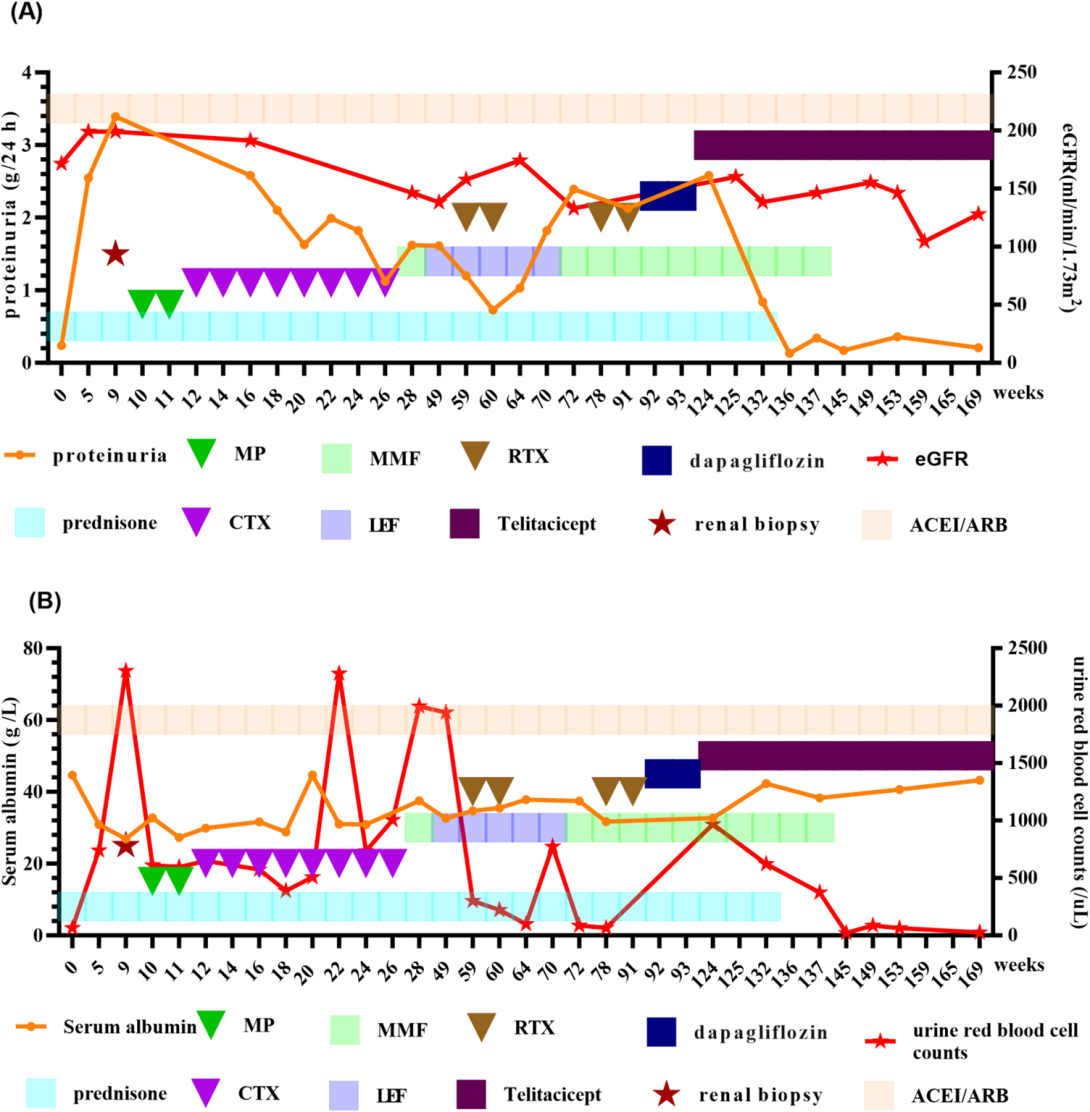
The relationship between follow-up data and treatments. MP: Pulses of intravenous methylprednisolone; CTX: Cyclophosphamide; MMF: Mycophenolate Mofetil; RTX: Rituximab; LEF: Leflunomide; eGFR: Estimated glomerular filtration rate

Subsequently, she underwent treatment with MP pulse therapy and intravenous CTX. Upon the completion of eight cycles of CTX treatment, amounting to a total dosage of 3.2 g, the child continued to display significant hematuria. Routine urine analysis revealed an elevated red blood cell count of 1005.4 /uL and a urine protein quantification of 1119.06 mg/24 h. Commencing in December 2021, CTX was stopped and MMF was administered orally in two doses, totaling 0.75 g/d, with the blood drug concentration kept within normal limits. MMF was orally administered for a duration of four months, during which the 24-h urine protein consistently surpassed 1000 mg. Considering the poor effect of MMF, the child went to another hospital for treatment and was treated with 10 mg/d of leflunomide (LEF) in May 2022. After two months of oral LEF administration, an improvement in hematuria was noted compared to the previous condition, with the urine red blood cell count decreasing to 300/μL. However, the urine protein quantification remained at 1014 mg/24 h. Despite undergoing a series of treatments with glucocorticoids and three immunosuppressive agents over a one-year period, the patient’s urinary protein quantification remained elevated, exceeding 1000 mg/24 h. Given the ineffectiveness of various prior drug therapies, it was noted that the peripheral blood CD19 + B cell counts were 393/μL. On July 18 and 25, 2022, RTX was administered twice at a dosage of 375 mg/m^2^, respectively. Subsequent to the RTX treatment, the child’s gross hematuria resolved, with the urinary protein quantification decreasing to 731.3 mg/24 h, and the peripheral blood CD19 + B cell counts decreasing to zero. Between August and October 2022, she continued to experience intermittent episodes of gross hematuria and repeated urinalysis indicated a urine protein level of 2 +  ~ 3 + . The child experienced two urinary tract infections accompanied by significant gross hematuria, during which time the serum immunoglobulin IgG level was measured to be 3.0 g/L, with the peak urine red blood cell count reaching 773/μL. After the administration of antibiotics, the symptoms of gross hematuria were alleviated. Subsequently, the patient received 20 g of immunoglobulin for immune support therapy, resulting in a significant reduction in the urine red blood cell counts to 64.9/μL. However, there was a recurrence in urinary protein quantification, which increased to 1823.1 mg/24 h. The child was then treated at the Children’s Hospital affiliated with Fudan University and restarted oral MMF at a dosage of 0.75 g/d combined with traditional Chinese medicine in December 2022. The amount of urinary protein continued to rise. Due to the consistent increase in the amount of urine protein, RTX was administered again on March 7th and 15th, 2023. However, RTX didn’t work very well. In July 2023, dapagliflozin was added at a dosage of 5 mg/d, in conjunction with oral MMF and fosinopril. During the entire medication period, renal function remained normal, but continuous microscopic hematuria accompanied by moderate to severe proteinuria was observed. The detailed medication process and the follow-up data are presented in Fig. 2.

**Fig. 3:**
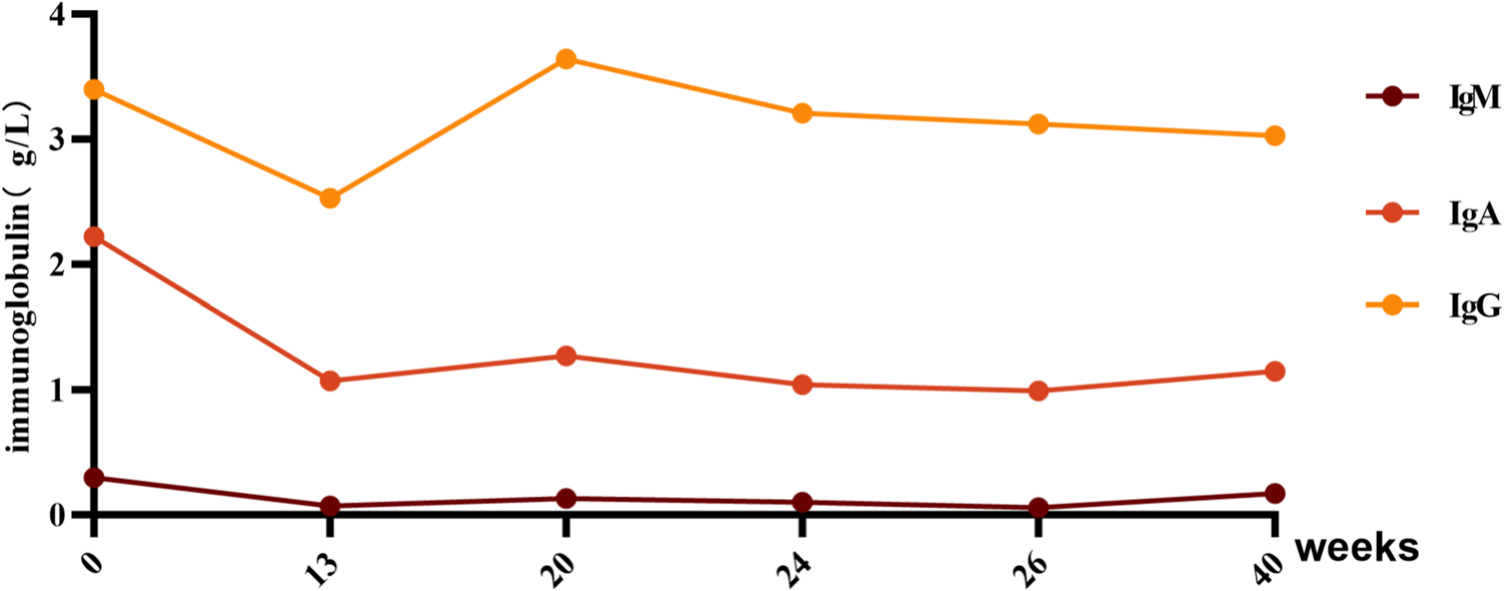
Follow-up of immunoglobulin levels

In October 2023, subsequent to an infection of the upper respiratory tract, the patient exhibited symptoms of hematuria, with a urine red blood cell counts elevating to 967.6/μL and a urinary protein quantification of 2582 mg/24 h. After a thorough evaluation to exclude any contraindications, treatment with telitacicept was initiated on October 21, 2023, at a dosage of 80 mg per injection, administered subcutaneously once weekly. After one month of telitacicept administration, the patient’s urinary protein quantification decreased to 837.9 mg/24 h, and the urine red blood cell counts decreased to 124.3/μL. By 12 weeks of telitacicept treatment, the patient’s routine urine test indicated that protein was negative. In January 2024, the patient was able to successfully reduce steroid dosage, and in April 2024, MMF was discontinued. After 10 months of telitacicept treatment, no significant gross hematuria was observed in the patient, and the urine red blood cell counts had decreased substantially to 27/μL. Furthermore, urine protein levels were effectively controlled and maintained within a range of 127 mg/24 h to 207 mg/24 h (Fig. 2). Specifically, the levels of immunoglobulin (IgA, IgM, IgG) did not show a significant decrease during the treatment with telitacicept (Fig. 3).

Upon initiating telitacicept therapy, the initial count of peripheral blood CD19 + B cells was documented as 1.63/μL. Following the administration of telitacicept, the monitored count of peripheral blood CD19 + B cells gradually increased, ultimately reaching a final count of 51.43/μL. Throughout the entire duration of treatment and subsequent follow-up, no life-threatening serious adverse events, including severe infections or anaphylactic shock, were noted. Throughout the course of the illness, the levels of blood creatinine, cystatin C, urine α1-microglobulin, and urine β2-microglobulin remained within normal range. Furthermore, the patient’s blood pressure and the renal function were also determined to be within normal ranges. As illustrated in Fig. 2, the patient’s eGFR decreased at week 25 following treatment with telitacicept, which was attributed to the viral gastroenteritis the patient experienced at that time. Given the presence of prerenal acute kidney injury during that period, the patient’s eGFR improved after the administration of fluid therapy. During the period of telitacicept therapy, the patient did not experience any weight gain, but did exhibit a growth in height by 2 cm.

## Discussion

In the present case, the patient failed to achieve clinical remission after treatment with glucocorticoids and various immunosuppressants. However, when treatment with telitacicept was introduced, proteinuria significantly decreased within a short period. The levels of immunoglobulin did not show a significant decrease during treatment with telitacicept, and no serious drug reactions or infections occurred. This case provides a new treatment approach for pediatric IgAVN.

The clinical features of IgAV and IgAN show notable similarities [13]. Key factors in the pathogenesis of IgAV include genetic predispositions, disrupted mucosal immunity, and immune complexes involving abnormal IgA or IgA antibodies [14]. Mucosal immunity, particularly within gastrointestinal lymphoid organs, may play a crucial role in the development of IgAV [14]. The inadequate therapeutic response to RTX observed in this case aligned with the findings of a recent clinical trial, which indicated that RTX did not effectively reduce proteinuria in patients with IgAN [15]. This is attributed to the fact that antibody production in patients with IgAN and IgAVN originates from mucosal and gut-associated lymphoid tissues [16, 17].

Targeting the B cell pathway to inhibit Gd-IgA1 and antibody production represents a therapeutic strategy grounded in the “quadruple whammy hypothesis” of IgAN pathogenesis [18, 19]. Quantification has demonstrated that elevated levels of Gd-IgA1 are associated with more severe pathological damage [20, 21]. Telitacicept, serving as a dual inhibitor of both BAFF and APRIL, functions to inhibit the production of pathogenic antibodies by B cells and plasma cells [10]. Jin et al. indicated that telitacicept reduced circulating levels of Gd-IgA1, IgG-IgA immune complexes, and poly-IgA immune complexes by approximately 50% [22]. Currently, telitacicept is utilized for the treatment of autoimmune diseases and is undergoing extensive clinical research globally to assess its efficacy in managing a wide array of autoimmune disorders [23]. Studies have shown that telitacicept may represent a safe and effective therapeutic option for IgAN, providing reductions in proteinuria and increases in eGFR comparable to conventional immunosuppressive (IS) therapy [24]. Therefore, we attempted to treat refractory IgAVN with telitacicept in this patient. After one month of telitacicept therapy, the child’s 24-h urinary protein level underwent a rapid decrease. Subsequent to 12 weeks of treatment, it reverted to normal levels. Upon discontinuation of glucocorticoids and MMF, the patient’s 24-h urinary protein levels continued to be consistently maintained at ≤ 200 mg/24 h.

The adverse reactions associated with telitacicept treatment were predominantly mild in nature. Despite existing literature documenting potential severe hypogammaglobulinemia in patients with systemic lupus erythematosus (SLE) following telitacicept administration, which may be accompanied by grave complications including encephalopathy, severe depression with suicidal ideation, and diffuse large B cell lymphoma [25], the immunoglobulin levels of the pediatric patient in question exhibited minimal variation during the course of telitacicept treatment (Fig. 3). It is noteworthy that no cases of severe infections have been reported. Additionally, there are documented cases of successful pregnancies in IgAN patients undergoing telitacicept therapy [26]. Furthermore, numerous clinical trials have confirmed the high safety profile of telitacicept with no notable side effects observed [24, 27].

In summary, this case serves as a clinical reference for the utilization of telitacicept in the treatment of refractory IgAVN. Although the precise pathogenesis of IgAVN remains unclear, targeting the production of Gd-IgA1 and the formation of its autoantibodies has emerged as a promising therapeutic strategy. This marks a shift in the management of IgAVN, transitioning from traditional immunosuppressive therapies to a targeted therapeutic approach grounded in an understanding of the underlying pathogenic mechanisms. As of the present moment, there have been no published reports on the application of telitacicept in IgAVN. Given that this is a single case study, it is challenging to conduct a comprehensive assessment of its long-term efficacy and safety. Therefore, we intend to conduct long-term and larger-scale clinical trials to further validate the therapeutic efficacy of telitacicept and investigate the potential relationship between alterations in the concentrations of immune complexes, such as Gd-IgA1, and treatment effectiveness.
